# Biological Evaluation and Molecular Docking Studies of Dimethylpyridine Derivatives

**DOI:** 10.3390/molecules24061093

**Published:** 2019-03-20

**Authors:** Piotr Świątek, Katarzyna Gębczak, Tomasz Gębarowski, Rafal Urniaz

**Affiliations:** 1 Department of Chemistry of Drugs, Faculty of Pharmacy, Wroclaw Medical University, Borowska 211, 50-556 Wroclaw, Poland; 2 Department of Basic Medical Science, Wrocław Medical University Borowska 211, 50-556 Wrocław, Poland; katarzyna.gebczak@umed.wroc.pl (K.G.); tomasz.gebarowski@umed.wroc.pl (T.G.); 3 Department of Medicine, Institute for Medical Research, University of Cambridge, Cambridge Biomedical Campus, Addenbrooke’s Hospital, Hills Rd, Cambridge CB2 0SP, UK; rafal.urniaz@gmail.com

**Keywords:** dimethylpyridine, anti-COX, molecular docking

## Abstract

Cyclooxygenase inhibitors as anti-inflammatory agents can be used in chemoprevention. Many in vitro and in vivo studies on human and animal models have explained the mechanisms of the chemopreventive effect of COX inhibitors such as: induction of apoptosis, inhibition of neoplasia, angiogenesis suppression, induction of cell cycle inhibition and inhibition of the expression of peroxisome proliferator-activated receptors. Here, biological evaluation of twelve different Schiff base derivatives of *N*-(2-hydrazine-2-oxoethyl)-4,6-dimethyl-2-sulfanylpyridine- 3-carboxamide are presented. Their in vitro anti-COX-1/COX-2, antioxidant and anticancer activities were studied. The molecular docking study was performed in order to understand the binding interaction of compounds in the active site of cyclooxygenases. Compounds PS18 and PS33 showed a significant inhibitory activity on COX-1 at lower concentrations compared to meloxicam and piroxicam. The IC_50_ of COX-1 of these compounds was 57.3 µM for PS18 and 51.8 µM for PS33. Out of the tested compounds, the highest therapeutic index was demonstrated by PS18, PS19, PS33, PS40 and PS41. Lower molar concentrations of these compounds inhibit the growth of cancer cells while not inhibiting the healthy cells. Compounds PS18, PS19 and PS33 simultaneously demonstrated a statistically-significant inhibition of COX-1 or COX-2. This opens up the possibility of applying these compounds in the chemoprevention of cancer.

## 1. Introduction

Cyclooxygenases (COX) are dual-function enzymes, which catalyse the conversion of arachidonic acid to prostaglandins and can occur in two isoforms: COX-1 and COX-2. COX-1 undergoes constitutive expression in most tissues and is involved in the production of prostaglandins (PG) in physiological intracellular processes. COX-2 is undetectable in most tissues under physiological conditions; however, it can be quickly, but temporarily, induced as a result of homeostatic disorders, e.g., osmosis disorders, inflammations [1]. The first generation of non-steroidal anti-inflammatory drugs (NSAIDs) inhibits the activity of COX in a non-selective manner and is associated with adverse effects, especially on the mucous membrane of the gastral and intestinal sections of the digestive tract. The discovery of COX-2 inhibitors (coxibs) made it possible to apply them primarily in the treatment of pain caused by neoplastic and non-neoplastic changes (e.g., rheumatoid arthritis, osteosclerosis). Unfortunately, this in turn was associated with adverse effects and increased the risk of cardiovascular diseases [2]. It is therefore unclear which degree of COX-2 selectivity should be considered safe. It appeared that developing moderately selective inhibitors rather than those possessing high selectivity might be a more balanced approach. Nevertheless, the identification of new inhibitors based on a novel scaffold possessing structural features other than that of the known inhibitors could be beneficial and desirable for the potential treatment of inflammatory diseases.

Many in vitro and in vivo studies on human and animal models explained such mechanisms of the chemopreventive effect of COX as induction of apoptosis, inhibition of neoplasia, angiogenesis suppression, induction of cell cycle inhibition and inhibition of the expression of peroxisome proliferator-activated receptors (PPARs) [3,4,5,6,7,8]. Studies on the preventive and therapeutic efficacy of COX inhibitors performed on rats induced with *N*-butyl-*N*-(4-hydroxybutyl)nitrosamine to cause bladder cancer have shown that COX inhibitors not only have a preventive effect on the occurrence and growth of neoplasm, but also affect the formation of preneoplastic changes [9]. It has been shown that administering the drug at a later time (after the induction of neoplasm) not only fails to inhibit the growth of the neoplasm, but also causes its development into a higher stage of malignancy and initiates further chains of inflammatory processes. The chemopreventive efficacy of a COX-2 inhibitor was obtained by using a lower dose compared with other studies [10]. The application of a low dose does not have a negative effect on the functioning of other organs, especially the stomach. Research on the chemopreventive effects of coxibs is also confirmed by earlier findings regarding the role of coxibs in the chemoprevention of colon cancer [11].

Oxidative stress and inflammation play a key role in the development of neoplasms, and the inhibition of these paths at an early stage of neoplasia may be a critical moment in the process of chemoprevention [12]. When the output of free radicals and active derivatives exceeds the body’s ability to neutralise and eliminate them, they become the cause of oxidative stress and may lead to DNA mutations. A lack of balance between the activity of prooxidants and antioxidants (lipoxygenase, cyclooxygenase and phospholipid-hydroperoxide glutathione peroxidase), hydroperoxides, lipid peroxidants, aldehydes and peroxynitrites is the main cause of oxidative stress. Inflammatory cells and cancer cells themselves produce free radicals, pro-inflammatory mediators such as cytokines, chemokines and arachidonic acid metabolites, which generate further production of reactive oxygen species (ROS), activating further series of inflammatory cells. ROS and reactive nitrogen species (RNS) may directly damage the DNA or interfere with the repair mechanisms thereof. They may also react with proteins, carbohydrates, as well as lipids, and the derived products may significantly disrupt the intracellular and extracellular homeostasis. On the other hand, other studies show that COX inhibitors are not only anti-inflammatory, but they also limit the production of free radicals [8,9]. The main substances that connect inflammation with cancer through oxidative/nitrosative stress are prostaglandins and cytokines. Chronic inflammations, and especially a multiplicity thereof, predispose susceptible cells to malignant transformation. The longer the inflammatory process, the greater the risk of cancer. Numerous studies show clear links between colorectal cancer and chronic inflammation (ulcerative colitis, Crohn’s disease), adenocarcinoma and reflux esophagitis (Barret’s oesophagus), hepatitis predisposing to liver cancer, schistosomiasis increasing the risk of bladder and colon cancer and a chronic *Helicobacter* infection leading to stomach cancer [13]. Epidemiological studies show a significantly lower risk of cancer in people who regularly use NSAIDs. Celecoxib is currently being tested as an anti-cancer drug in clinical trials. It regulates the number of colon polyps in cases of familial adenomatous polyposis [14]. It is therefore important to determine the most rational and effective combination of redox-active and anti-inflammatory compounds with other standard anti-cancer drugs and to identify a low, non-toxic dose thereof.

For years, research has been conducted on new compounds of analgesic and anti-inflammatory properties. Scientists are very interested in compounds with an acylhydrazone or arylhydrazone group. Many authors suggest that it is a pharmacophoric group responsible for the analgesic activity of compounds [15,16,17]. Mahy et al. proposed a mechanism of N-arylhydrazone compounds’ activity, which is probably based on cyclooxygenase inhibition [18]. The benzo-bis-aza allylic fragment, derived from aryl- or acyl-hydrazone, as well the bis-allylic methylene fragment in arachidonic acid show isosteric similarity, thanks to which the hydrazone derivative is able to block cyclooxygenase as a fake ligand, thus blocking the enzymatic cascade of arachidonic acid and the production of pro-inflammatory factors [15,19].

## 2. Results and Discussion

### 2.1. Chemistry

**Figure 1:**
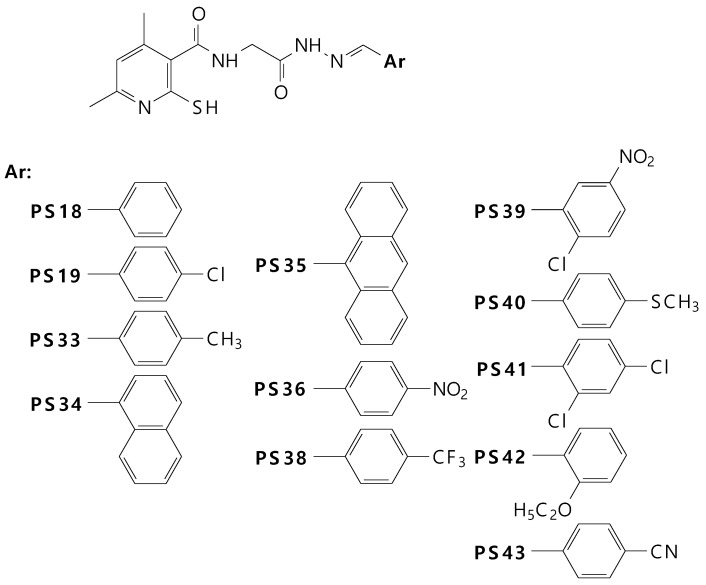
New derivatives of dimethylpyridine.

The structures of the derivatives specified in the title and used in the experiments are presented in Figure 1. The synthesis of dimethylpyridines was described previously [20]. The synthesis of twelve different Schiff base derivatives of *N*-(2-hydrazine-2-oxoethyl)- 4,6-dimethyl-2-sulfanylpyridine-3-carboxamide was carried out by the reaction of this compound with several aromatic aldehydes in methanol in the presence of a catalytic amount of acetic acid with very good yields. The purity of the synthesized compounds was checked by elemental analyses. The structures of the synthesized compounds were determined based on spectral data analysis, such as IR and ^1^H-NMR.

### 2.2. Biological Evaluation

#### 2.2.1. Anti-COX Activity

The compounds were investigated for their potencies to inhibit COX-1 and COX-2 enzymes by the colorimetric inhibitor screening assay.

IC_50_ values were calculated (i.e., the concentration of tested compounds (µM) that can exert 50% inhibition of the enzyme activity), separately with COX-1 and COX-2 activity estimations at 2 min of incubation with the tested compounds. Selectivity of the compounds to COX-1 or to COX-2 was assessed by calculation of the IC_50_ ratios. The IC_50_ values were not calculated with those tested compounds that exerted very low inhibitory activity at 2 min of incubation. The results of the calculation are given in Table 1.

As can be seen from the data presented in Table 1, the studied compounds PS34, PS35, PS38, PS39 and PS41 demonstrated no inhibitory activity on either COX-1 or COX-2. P36 showed statistically-significant inhibitory activity on COX-2 at a higher concentration in comparison with piroxicam or meloxicam. It did not show any inhibitory activity on COX-1.

The PS19 (*N*′-4-chlorophenylmethylene acetohydrazide), PS40 (*N*′-4-methylthiophenylmethylene acetohydrazide) and PS43 (*N*′-4-cyanophenylmethylene acetohydrazide) compounds demonstrated a statistically-significant inhibitory activity on COX-1 at lower concentrations compared with piroxicam. No inhibitory activity on COX-2 occurred apart from PS43, which retained a COX-2 inhibitory activity at piroxicam’s level.

PS18 (*N*′-phenylmethylene derivative) and PS33 (*N*′-4-methylphenylmethylene derivative) showed a statistically-significant increased inhibitory activity on COX-1 at lower concentrations compared to meloxicam and piroxicam. The IC_50_ of COX-1 of these compounds was 57.3 µM for PS18 and 51.8 µM for PS33. Meanwhile, they demonstrated a COX-2 inhibitory activity at piroxicam’s level (no significant differences) and a significantly inhibitory activity on COX-2 at higher concentrations compared with meloxicam.

The compound PS42 inhibited in a statistically-significant manner the activity of COX-1 (at a lower concentration) and COX-2 (at a higher concentration) compared with the inhibitory activity of piroxicam and meloxicam. 

In the case of COX-2, none of the examined compounds reached 50% of inhibitory activity at lower concentrations compared with both piroxicam and meloxicam control compounds.

Data on the ratios of COX-2 to COX-1 activity are included in Table 1 clearly indicates that compounds PS18, PS33, PS42 and PS 43 exerted significant COX-1 selectivity in contrast to reference drugs.

#### 2.2.2. Antiradical Activity

Studies conducted on rats have shown that overexpression and increased activity of COX-2 were induced by reactive oxygen species as a result of mechanical stress (unilateral ureteral injury) and oxidative stress (treatment of the renal core interstitial lines (RRMICs)). Other studies indicate that COX-2 is the target in response to pro-inflammatory factors to induce and maintain endothelial dysfunction in hypertension and diabetes [21,22]. COX-2 are known to promote oxidative stress and vascular dysfunction [23]. Taking into account these reports, the antiradical activity of new dimethylpyridine derivatives was investigated. The results of the test are summarized in Table 2. 

In the case of compound PS34 (*N*′-naphtylmethylene derivative), the number of free radicals under static conditions (no oxidative stress induced by hydrogen peroxide) decreased in a statistically-significant manner compared with the control group. Under oxidative stress induced by the addition of hydrogen peroxide, the studied PS18, PS34, PS35, PS36, PS39, PS40, PS41, PS42 and PS43 showed a statistically-significant free radical activity decrease compared with the control group. The tested compound PS34 reduced the number of free radicals, both under stable conditions and under oxidative stress.

#### 2.2.3. Cytotoxicity

The sulforhodamine B assay was used to estimate the cytotoxic properties of the compounds. The cytotoxic activity of dimethylpyridine derivatives was studied against human cancer cell lines: A549 (pulmonary basal cell alveolar adenocarcinoma) and LoVo (colon adenocarcinoma). Additionally, the effect of compounds on NHDF (normal human dermal fibroblasts) and normal cell line V79 (fibroblast from Chinese hamster lung) was examined. The results obtained are presented in Table 3.

Out of the tested compounds, the highest therapeutic index, i.e., the difference between the concentrations that inhibit 50% of healthy and cancerous cells, was demonstrated by PS18, PS19, PS33, PS40 and PS41. Lower molar concentrations of these compounds inhibited the growth of cancer cells, while not inhibiting the healthy cells. In the case of compounds PS19, PS40 and PS41, the IC_50,_ values for tumour cells were 2–4-times lower than for normal cells.

Compounds PS18, PS19 and PS33 simultaneously demonstrated a statistically-significant inhibition of COX-1 or COX-2. This opens up the possibility of applying these compounds in the chemoprevention of cancer. Further research on compounds demonstrating both chemopreventive and anti-inflammatory properties is particularly important. Its importance stems from the fact that neoplasms are accompanied by inflammations that are often able not only to further the growth of already existing neoplasms, but also produce neoplasms of all kinds. Such is the case of stomach cancer, which develops as a result of inflammation caused by *Helicobacter pylori*. Over time, hepatitis B can develop into hepatocellular carcinoma, while prostatitis may cause prostate cancer.

### 2.3. Molecular Modelling Studies

For the dimethylpyridine derivatives, the mode of binding to COX-1 and COX-2 was characterized by a molecular docking study. The docking results and biological activity expressed as pIC_50_ are presented in Table 4.

**Figure 2:**
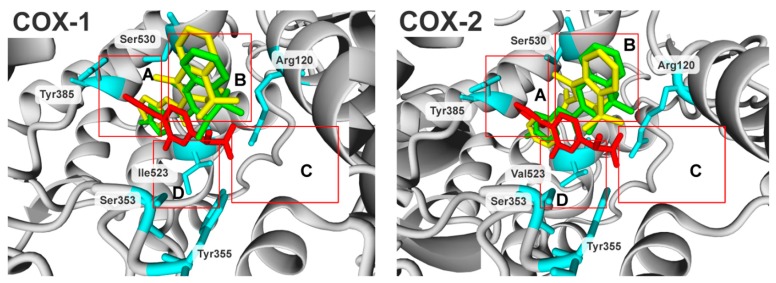
Illustration of four characteristic subdomains of the COX enzyme ligand-binding domain, indicated by red boxes, A, B, C and D. The flurbiprofen, meloxicam and piroxicam are coloured in red, green and yellow, respectively.

In terms of molecular biology, the enzymes have near-identical molecular weight and catalytic sites, although their amino acid sequence homology is only 65%, which implies a slightly different mode of binding for the same compounds under COX-1 and COX-2 binding conditions [24,25,26,27,28]. Previously, in the COX ligand-binding domain, the authors specified four characteristic subdomains of the enzyme, presented in Figure 2 as red boxes, A, B, C and D. 

The subdomains represent pockets where the native substrate (arachidonic acid), as well as the enzyme inhibitors may potentially bind. The subdomains were described in detail elsewhere [24] and will be summarized briefly. Three of the inhibitors, flurbiprofen, meloxicam and piroxicam, representing high biological activity, have been shown to facilitate the orientation of the subdomains. Subdomain A represents the mode of binding of flurbiprofen; subdomain B represents the mode of binding of meloxicam and piroxicam; subdomain C represents an entrance region of the enzyme binding domain; and subdomain D represents the position of the residue in position 523; the isoleucine in COX-1 and the valine in COX-2. Position 523 is the main cluster of differentiation between COX-1 and COX-2 active sites, being the main target for selective compounds. The smaller Val523 residue in COX-2 allows access to a hydrophobic side-pocket in the enzyme, which is sterically hindered by Ile523 [29]. The protein surfaces (Supporting Information, Figure S1) and binding cavities in terms of electrostatic potential (Supporting Information, Figure S2) and hydrophobicity (Supporting Information, Figure S3) are compared in the Supporting Information. 

To correlate the mode of binding with biological activity, the compounds were docked to the corresponding crystal structures and evaluated by the scoring function. The selected docking results are compiled in Table 4. Full results are presented in the Supporting Materials in Table S1.

The docking study showed that compounds might be divided into three different groups referring to the preferred (meaning low energy) docking positions. The first group of compounds (PS34, PS35, PS36, PS38, PS41 for COX-1 and PS19, PS34, PS36, PS38, PS39, PS40, SP41 for COX-2) docked outside of the binding cavity; the second explored the binding cavity partially (PS35, PS39 for COX-1 and PS35 for COX-2); and the third docked into the regular binding domain (PS18, PS19, PS33, PS40, PS42, PS43 for COX-1 and PS18, PS33, PS42, PS43 for COX-2), as can be seen by the example of PS34, PS43 and PS35 in Figure S4 in the Supporting Information.

**Figure 3:**
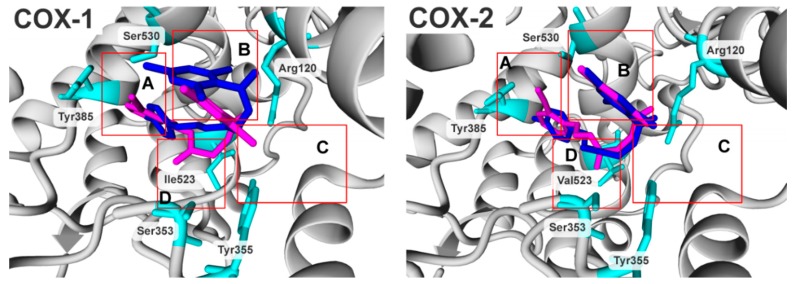
Docking poses of PS33 (blue) and PS43 (magenta) under COX-1 and COX-2 binding domain conditions. Residues coloured cyan; all hydrogens were hidden. A, B, C, D: binding subdomains corresponding to Figure 2; details are given in the text.

When the biological activity of compounds was compared (Table 4, pIC_50_), the most potent compounds were PS33 in the COX-1 group and PS43 in the COX-2 group. Additionally, PS33 had the highest selectivity ratio in the presented cohort of compounds. The compounds share the same orientation in both binding cavities where the dimethylpyridine is directed into subdomain B (Figure 3, B) and the aryl moiety is directed into subdomain A (Figure 3, A). Subdomain A represents the mode of binding of flurbiprofen, and subdomain B represents the mode of binding of meloxicam and piroxicam.

The two compounds are able to explore both (A and B) subdomains and benefit from the mode of binding of flurbiprofen, meloxicam and piroxicam, which is reflected in biological activity. They act in a lower concentration comparing to the meloxicam and piroxicam (Table S1, Supporting Information). Additionally, as presented in Figure S5 in the Supporting Information, both compounds interact by hydrogen bonds with Arg120, Ala527 and Leu531 in the case of PS33 and Arg120, Tyr355 and Ser530 in the case of PS43 under COX-1 binding conditions and Arg120 and Tyr355 in the case of PS33 and Arg120 and Ser530 in the case of PS43 under COX-2 binding conditions. 

Compound PS43 (Figure 3, magenta) had almost the same position under COX-1 and COX-2 binding conditions. However, it gained stability by the steric interaction with valine 523 under COX-2 (Figure 3, D), and this interaction was lost under COX-1. The PS33 did not benefit from the steric interaction (Val 523 in COX-2 nor Ile523 in COX-1), although it can benefit from strong hydrogen bond interactions with the arginine 120 (Arg120) and had better overall steric fit under COX-1 binding conditions, which was lost under COX-2. It had a direct influence on the compound selectivity and biological activity under the COX binding conditions. 

It is well recognised that steric fit and hydrogen bond interactions are among the most important components that reflect biological activity in the case of enzyme inhibition [30]. Hood et al. [31] underlined the importance of the charged arginine 120 residue located at the entrance to the main hydrophobic channel. Other studies showed that the main interaction of COX inhibitors with enzyme is mostly by hydrogen bond and hydrophobic interactions with the binding pockets [25,26,27,28]. Previously-reported results and our observation explain the behaviour of the presented compounds and their biological activity.

## 3. Materials and Methods

### 3.1. Chemicals

Sulforhodamine (SRB), Trizma-base and trichloroacetic acid (TCA) were purchased from Sigma-Aldrich (St. Louis, MO, USA). Foetal bovine serum (FBS), 2 mM L-glutamine solution, antibiotic solution (penicillin (100 U/mL) and streptomycin (0.1 mg/mL)) and trypsin EDTA solution were obtained from Lonza (Verviers, Belgium). Cell culture plastic flasks (75 cm^2^), as well as 96-well culture plastic plates were purchased from Lonza (Verviers, Belgium).

Phosphate-buffered saline (PBS) and 0.4% trypan blue solution were obtained from POCH (Gliwice, Poland).

### 3.2. Cells

Normal human dermal fibroblasts (NHDF) from adult donors were purchased from Lonza (Verviers, Belgium). Normal cell line V79 (fibroblast from Chinese hamster lung) and human cancer cell lines, A549 (pulmonary basal cell alveolar adenocarcinoma) and LoVo (colon adenocarcinoma) were obtained from the European Collection of Authenticated Cell Cultures (ECACC).

### 3.3. Methods

#### 3.3.1. COX Colorimetric Inhibitor Screening Assay Kit (Cayman Chemical Company, Ann Arbor, MI, USA)

This method allows one to estimate the peroxidase activity of COX by colorimetric monitoring of the occurrence of the oxidized form of *N*,*N*,*N*′,*N*′-tetramethyl-*p*-phenylenediamine (TMPD) at 590 nm. TMPD is a substrate for most enzymes with peroxidase activity, and high throughput microplate assays using TMPD allow the rapid screening of a wide range of therapeutics that inhibit COX activity in vitro.

The test is based on the oxidation of TMPD during the reduction of PGG2 (prostaglandin G2) to PGH2, which is reflected by a change in colour, measured spectrophotometrically (Victor2 microspectrophotometer, PerkinElmer Waltham, MA, USA). The assay uses Tris-HCl buffer (0.1 M assay buffer, pH 8.0), a solution of heme in dimethylsulfoxide (DMSO), enzymes (COX-1, COX-2), arachidonic acid (100 μM), KOH (0.1 M) and a solution of TMPD. The assay mixture contains: 150 µL of assay buffer, 10 µL of heme and 10 µL of COX-1 or COX-2. In order to determine 100% enzyme activity (each COX sample was assayed in triplicate), 10 µL of the substances used as solvents (methanol, ethanol, DMSO) was added to the wells. Ten microlitres of tested inhibitors at appropriate concentrations were added to the other wells. Twenty microlitres of TMPD were added to all wells. The reaction was initiated by the addition of arachidonic acid.

The effect of tested inhibitors on COX-1 and COX-2 enzyme activity was measured by assaying the rate of TMPD oxidation within 2 min in a spectrophotometer at 590 nm.

The activity factor at 2 min of incubation with the tested compounds in comparison to the initial activity of the enzyme was determined. This enabled the calculation of IC_50_ values (concentrations at which 50% inhibition of enzyme activity occurred).

#### 3.3.2. Cell and Culture Conditions

Cells were grown in the culture media recommended by the cell line supplier. Before the test, adherent cells were detached with the trypsin EDTA solution, and FBS-containing medium was used to neutralize the effects of the trypsin/EDTA solution. After that, cells were spun down, counted, stained with a 0.4% solution of trypan blue and inspected under a microscope for cell viability. Then, cells were placed on 96-well plastic culture plates (2 × 10^3^ cells/well) and incubated at 37 °C in a CO_2_-incubator for 24 h. Afterwards, all the tested compounds were added at appropriate final concentrations (100 µM, 50 µM, 20 µM, 10 µM, 5 µM), and the cultures were incubated for 48 h in CO_2_-incubator at 37 °C. Finally, cells were harvested and intended for the cell proliferation test.

#### 3.3.3. Determination of Cell Density/Cell Proliferation

Cell density/cell proliferation was estimated with the sulforhodamine B (SRB)-colorimetric assay [32]. Briefly, cell cultures were fixed with cold TCA (final concentration 10% (*w*/*v*) in cultures of adherent cells for 1 h at 4 °C, then washed four times with tap water and air-dried at room temperature (20–25 °C). A mildly-acidic SRB solution (0.4% dye solution in 1% acetic acid) was added to each well for 30 min at 25 °C. Then, the unbound stain was removed by rinsing with an aqueous solution of 1% (*v*/*v*) acetic acid. Culture plates were then allowed to dry at room temperature. The protein-bound dye was dissolved in 10 mM Tris base solution (pH 10.5) for 10 min on a gyratory shaker. Absorbance of the SRB solution was estimated at 540 nm in a Victor 2 microplate reader (Perkin-Elmer, Waltham, MA, USA).

#### 3.3.4. Evaluation of Intracellular ROS Level

The cell-permeable, fluorescence probe DCFH-DA (2′,7′-dichlorodihydrofluorescein diacetate, final concentration of 25 μM) was added to the cell culture for the last 2 h of culture in the dark at 37 °C in the CO_2_ incubator, according to the standard procedure [33]. Then, the cells were washed twice with PBS and treated with H_2_O_2_ (100 μM) for 30 min. The fluorescence of dichlorodihydrofluorescein was measured (λex. = 485 nm, λem. = 535 nm) in the Victor2 microspectrophotometer (PerkinElmer, Waltham, MA, USA). The applied concentration of H_2_O_2_ (100 μM) was within the range of levels naturally present in skin wounds (50–200 μM) [34]. Thirty-minute cell incubation with H_2_O_2_ was chosen, as data found in the literature strongly suggest that cells were able to decompose almost all the H_2_O_2_ in the culture medium within 30 min [35].

#### 3.3.5. Statistical Analysis

The statistical significance of the results was calculated with the paired *t*-test and with two-way analysis of variance (ANOVA), following the routine statistical methods.

#### 3.3.6. Molecular Docking

The Protein Data Bank (PDB) database contains more than 81 crystal structures of two isozymes of the cyclooxygenase, and a number of crystal structures were co-crystallized with ligands. The complete, high-resolution X-ray structures of murine cyclooxygenase crystallized for Cox-1 (PDB ID: 4O1Z) and Cox-2 (PDB ID: 4M11) variants were chosen for molecular docking due to the absence of the human enzymes. Both structures were co-crystallized with the same ligand (meloxicam). The sequence identity compared to human variants is very high (~95%), and the residues comprising the active sites are fully conserved, as described elsewhere [30]. Primarily, the protein models were verified in terms of proper bond types and chemical structure. All cofactors were removed. Subsequently, the structures were aligned using a multiple structural alignment algorithm (MUSTANG) implemented in Yasara 11.6.16.33. to unify the coordinates. The program constructs a multiple alignment using the spatial information of the α-carbon atoms of the protein in the set. The ligands were built de novo in Spartan’10, and the Hartree−Fock ab initio algorithm with the 6-31G* basis set was used to minimize the energies. The compounds were exported as mol2 files and docked into the crystal structure. Molegro Virtual Docker 6.0 was chosen for the docking study as successfully applied by other investigators [24,36,37,38,39]. Moreover, the docking performance was described in the supporting materials elsewhere [24]. If not marked, the default settings were applied; the calculations were parameterized as follows: blind docking to whole binding domain, MolDock Score [GRID] searching and scoring algorithm, grid resolution 0.3 A., radius 15, search area: x: 63.25 y: 23.03 z: 206.04, number of iterations: 1500, number of runs: 1000, max number of poses return: 10, max population size 50.

## 4. Conclusions

In this study, biological evaluation of the series of twelve Schiff bases was presented. Their in vitro anti-COX-1/COX-2, antioxidant and anticancer activities were studied. A molecular docking study was performed in order to understand the binding interaction of the compounds in the active site of cyclooxygenases. The *N*′-phenylmethylene derivative PS18 and *N*′-4-methylphenylmethylene derivative PS33 showed a statistically-significant increased inhibitory activity on COX-1 at lower concentrations compared to meloxicam and piroxicam. In addition, the compound PS43 inhibited COX-2 activity at a similar level as piroxicam. It is worth noting that in the tests of antiradical activity, compound PS34 reduced the number of free radicals both under stable conditions and under oxidative stress. In the cytotoxicity tests, the highest therapeutic index was demonstrated by PS18, PS19, PS33, PS40 and PS41. Lower molar concentrations of these compounds inhibited the growth of cancer cells while not inhibiting the healthy cells. In the case of compounds PS19, PS40 and PS41, the IC_50_ values for tumour cells were 2–4-times lower than for normal cells. Compounds PS18, PS19 and PS33 simultaneously demonstrated a statistically-significant inhibition of COX-1 or COX-2. This opens up the possibility of applying these compounds in the chemoprevention of cancer.

## Figures and Tables

**Table 1:** COX-1 and COX-2 IC_50_ (SD) values for the studied compounds as compared with piroxicam and meloxicam (mean (SD); *n* = 3).

	IC_50_ (µM),(SD)		Ratio: COX-2/COX-1
Compound	COX-1	COX-2	
Meloxicam	85.8 (12.5)	71.5 (5.1)	0.8
Piroxicam	170.5 (15.7)	127.6 (11.9)	0.7
**PS18**	57.3 (1.1)*Δ	139.5 (15.7) Δ	2.4
PS19	92.9 (4.4) *	NA	-
**PS33**	51.8 (0.6) *Δ	142.9 (22.9) Δ	2.8
PS34	NA	NA	-
PS35	NA	NA	-
PS36	NA	730.7 (202.8) *Δ	-
PS38	NA	NA	-
PS39	NA	NA	-
PS40	83.6 (13.5) *	NA	-
PS41	NA	NA	-
PS42	72.8 (33.8) *	176.7 (14.3) *Δ	2.4
**PS43**	78.8 (5.5) *	**132.2 (128.1)**	1.7

Paired sample *t*-test for the evaluation of COX-1 and COX-2 activity compared to the control compound piroxicam (* *p* < 0.05) and the control compound meloxicam (Δ *p* < 0.05).

**Table 2:** Comparison of the free radical activity of the compounds studied (100 µM) using DCFH-DA (2′,7′-dichlorodihydrofluorescein diacetate) (mean (SD); *n* = 3).

E/E0	Without H_2_O_2_			With H_2_O_2_		
Compound	Average	Standard Deviation	*p*	Average	Standard Deviation	*p*
Meloxicam	0.99	0.20	NS	1.22	0.14	NS
Piroxicam	1.19	0.17	NS	1.59	0.25	NS
PS18	0.87	0.08	NS	0.70	0.06	0.01 *
PS19	0.84	0.12	NS	0.72	0.06	NS
PS33	0.86	0.09	NS	0.80	0.16	NS
**PS34**	**0.73**	**0.03**	0.004 *	**0.58**	**0.07**	0.01 *
PS35	1.07	0.28	NS	0.84	0.05	0.03 *
PS36	1.05	0.29	NS	0.75	0.06	0.02 *
PS38	1.10	0.29	NS	0.89	0.05	NS
PS39	1.06	0.24	NS	0.79	0.05	0.02 *
PS40	1.00	0.26	NS	0.67	0.04	0.004 *
PS41	0.95	0.21	NS	0.53	0.04	0.003 *
PS42	1.00	0.24	NS	0.59	0.01	0.0002 *
PS43	1.14	0.28	NS	0.77	0.02	0.003 *

* Statistical significance evaluated by a paired sample *t*-test (*p* < 0.05).

**Table 3:** Normal human dermal fibroblast (NHDF), LoVo and V79 IC_50_ (SD) values for the studied compounds (mean (SD); *n* = 3).

IC_50_ (µM) (SD)				
Compound	NHDF	V79	LoVo	A549
Meloxicam	205.6 (44.2)	231.8 (33.5)	124.6 (11.2)	148.3 (37.9)
Piroxicam	170.5 (23.0)	200.0 (32.9)	122.1 (9.6)	138.1 (27.8)
**PS18**	**171.4 (6.9)**	**275.0 (19.5)**	**135.3 (7.5)**	**154.2 (28.8)**
**PS19**	**213.9 (48.4)**	**338.5 (151.3**)	**115.1 (7.3)**	**163.9 (22.9)**
**PS33**	**159.1 (3.6)**	**177.9 (30.4)**	**130.9 (8.6)**	**156.3 (12.6)**
PS34	NA	420.9 (145.6)	NA	NA
PS35	202.4 (32.6)	196.2(76.2)	211.9 (76.4)	NA
PS36	143.7 (7.9)	140.6 (26.7)	99.8 (7.9)	163.5 (33.7)
PS38	NA	171.6 (36.2)	NA	503.8 (171.4)
PS39	391.0 (144.6)	146.8 (10.3)	103.0 (6.6)	119.7 (12.8)
**PS40**	**NA**	**608.2 (287.5)**	**NA**	**147.2 (22.3)**
**PS41**	**335.7 (118.3)**	**418.1 (349.6)**	**153.9 (12.2)**	**220.4 (47.8)**
PS42	132.4 (11.6)	162.0 (21.7)	133.2 (24.2)	139.9 (11.0)
PS43	177.1 (39.3)	255.8 (8.2)	258.5 (183.5)	221.5 (53.1)

**Table 4:** Selected docking results sorted by biological activity for COX-1 and COX-2, respectively.

Name	COX-1		Name	COX-2	
	pIC_50_	MolDock Score		pIC_50_	MolDock Score
**PS33**	**4.29**	−143.779	**PS43**	**3.88**	−141.132
PS18	4.24	−132.919	PS18	3.86	−130.663
PS 42	4.14	−129.366	PS33	3.84	−130.689
PS43	4.10	−127.159	PS42	3.75	−129.073

pIC_50_—the negative logarithm of the half maximal inhibitory concentration (IC_50_).

## Supplementary Materials

The following materials are available online. Figure S1. Protein surface of COX-1 and COX-2 coloured according to the electrostatic potential (red- and blue-coloured areas correspond to regions with respectively negatively- and positively-charged residues). Figure S2. Surface of COX-1 and COX-2 binding domain coloured according to the electrostatic potential. Red- and blue-coloured areas correspond to regions with respectively negatively- and positively-charged residues; (a) the view refers to Figure 2; (b) the view rotated 90° compared to the position of (a); the red box refers to Figure 2; the C box represents the entrance to the protein biding side; the red dotted line at COX-2 corresponds to the same position at COX-1. Figure S3. Surface of COX-1 and COX-2 binding domain coloured according to the hydropathy index proposed by Kyte and Doolittle. Hydrophilic residues are coloured red; hydrophobic residues are coloured blue; (a) the view refers to Figure 2; (b) the view rotated 90° compared to the position of (a); the red box refers to the C box of Figure 2 and represents the entrance to the protein biding side; the red dotted line of COX-2 corresponds to the same position of COX-1. Table S1: Results and biological activity for the COX-1 and COX-2 docking study. Figure S4. An overview of the docking positions for PS33 (light blue), PS43 (magenta) and PS35 (yellow) under the COX-1 and COX-2 binding conditions. The flurbiprofen (red) shows facilitation of the orientation in the binding cavity. Figure S5. Docking poses of PS33 (blue) and PS43 (magenta) under COX-1 and COX-2 binding domain conditions. Ligand maps representing interactions by hydrogen bonds (blue dot line); details are given in the main text.
