# Supplementary material for: Biological Evaluation and Molecular Docking Studies of Dimethylpyridine Derivatives
Source: Molecules. 2019 Mar 20;24(6):1093. doi: 10.3390/molecules24061093 (PMC6471528; doi:10.3390/molecules24061093)
Supplement: Supplementary file 1 [file molecules-24-01093-s001.pdf]

Supporting information

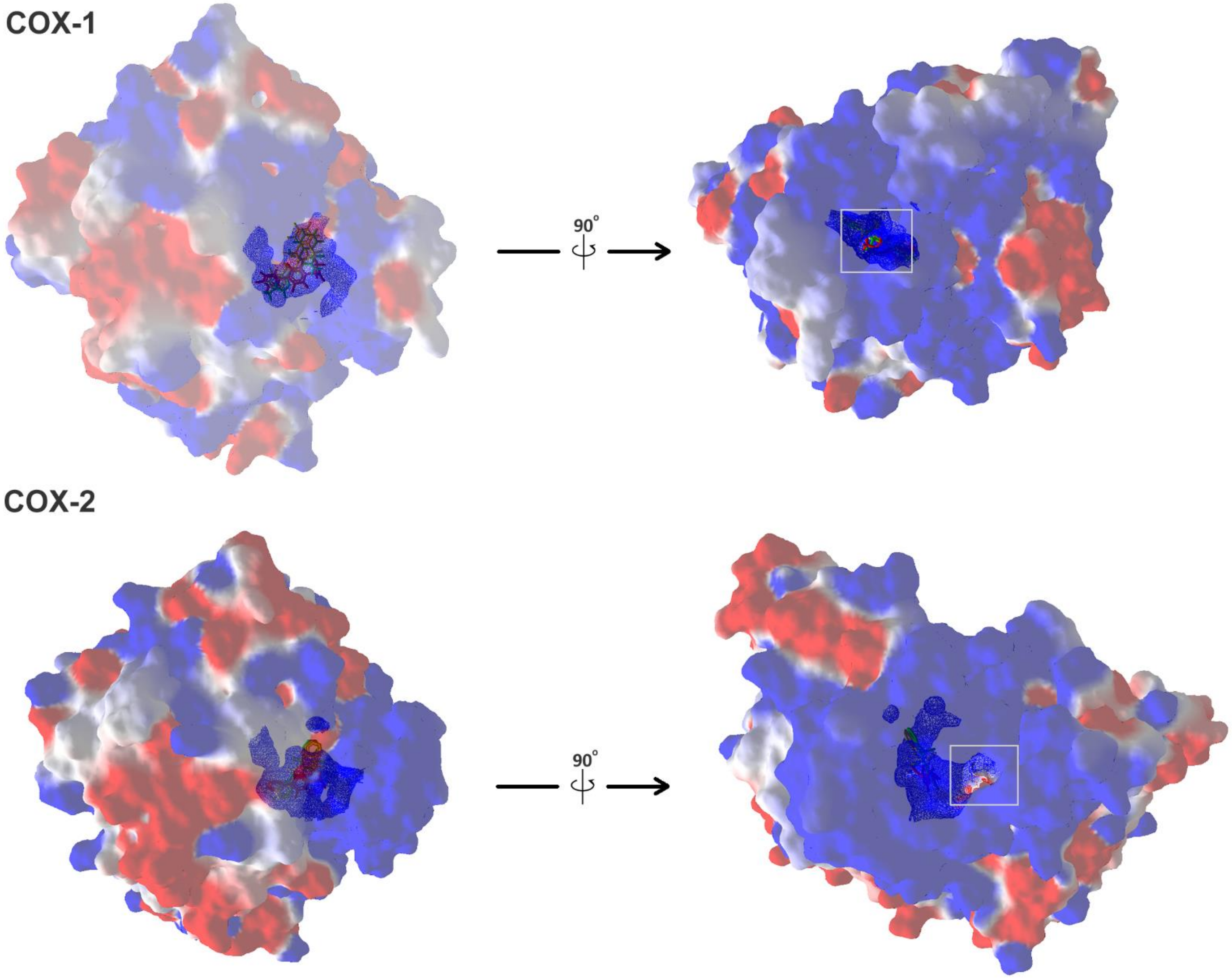

Figure S1. Protein surface of COX-1 and COX-2 coloured according to the electrostatic potential (red and blue coloured areas correspond to regions with respectively negatively and positively charged residues).

Supporting information

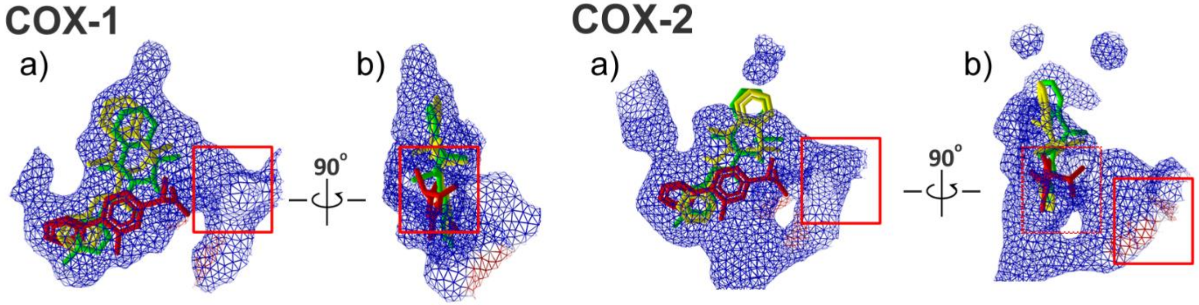

Figure S2. Surface of COX-1 and COX-2 binding domain coloured according to the electrostatic potential. Red and blue coloured areas correspond to regions with respectively negatively and positively charged residues; a) view refers to Figure 2; b) view rotated 90° comparing to a) position; red box refers to Figure 2, C box and represents entrance to the protein biding side; red dotted line at COX-2 corresponds to the same position at COX-1.

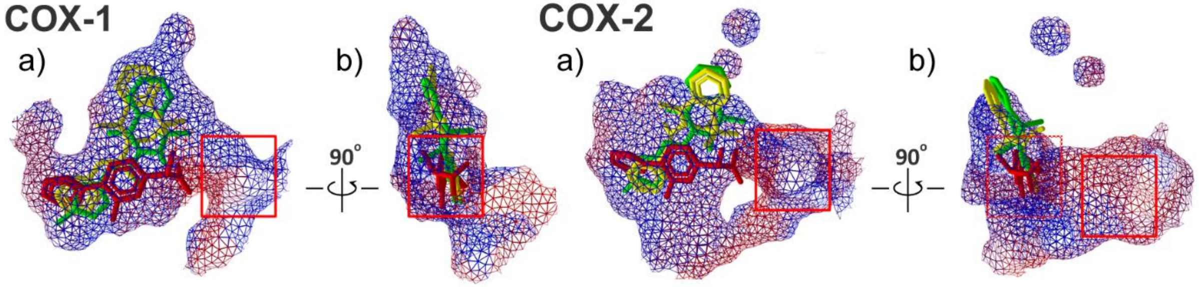

Figure S3. Surface of COX-1 and COX-2 binding domain coloured according to the hydropathy index proposed by Kyte and Doolittle (Kyte, J.; Doolittle, R.F. A Simple Method for Displaying the Hydropathic Characterofa Protein. *J. Mol. Biol.* **1982**, *157*, 105–132. doi:10.1016/0022-2836(82)90515-0). Hydrophilic residues are coloured red, hydrophobic residues are coloured blue; a) view refers to Figure 2; b) view rotated 90° comparing to a) position; red box refers to C box at Figure 2 and represents entrance to the protein biding side; red dotted line at COX-2 corresponds to the same position at COX-1.

Supporting information

Table S1. Results and biological activity for COX-1 and COX-2 docking study.

Name	COX-1		COX-2	
	pIC_{50}	MolDock S[illegible]	pIC_{50}	MolDock S[illegible]
PS33	4.29	-143.78	3,84	-130.689
PS18	4.24	-132.92	3,86	-130.66
PS 42	4.14	-129.37	3,75	-129.07
PS43	4.10	-127.16	3,88	-141.132
PS40	4.08	-118.49	NA	-132.22
**Meloxicam**	**4.07**	**-86.539**	**4,15**	**-88.866**
PS19	4.03	-107.393	NA	-131.87
**Piroxicam**	**3.77**	**-101.59**	**3,89**	**-37.678**
PS34	NA	-127.09	NA	-141.1
PS35	NA	-136.87	NA	-145.3
PS36	NA	-115.69	3,14	-138.36
PS38	NA	-125.5	NA	-128.53
PS39	NA	-131.71	NA	-136.21
PS41	NA	-116.04	NA	-138.14

Supporting information

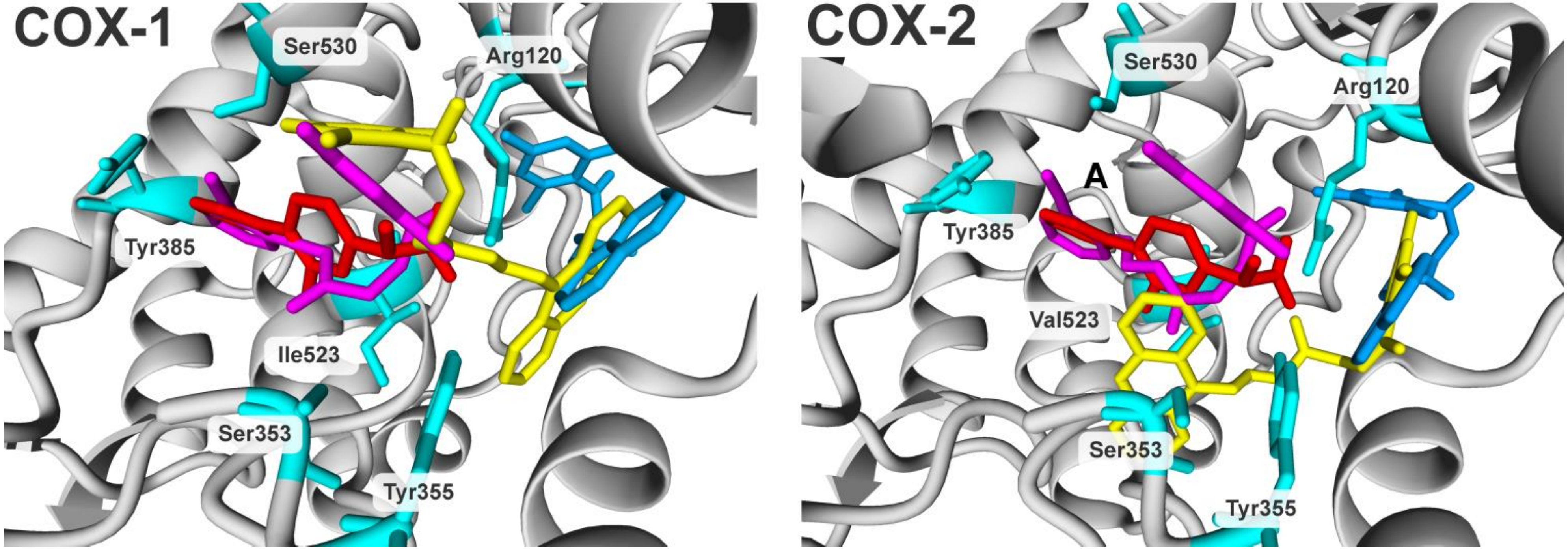

Figure S4. An overview of docking positions for PS34 (light blue), PS43 (magenta) and PS35 (yellow) under the COX-1 and COX-2 binding conditions. The flurbiprofen (red) show to facilitate the orientation in the binding cavity.

Supporting information

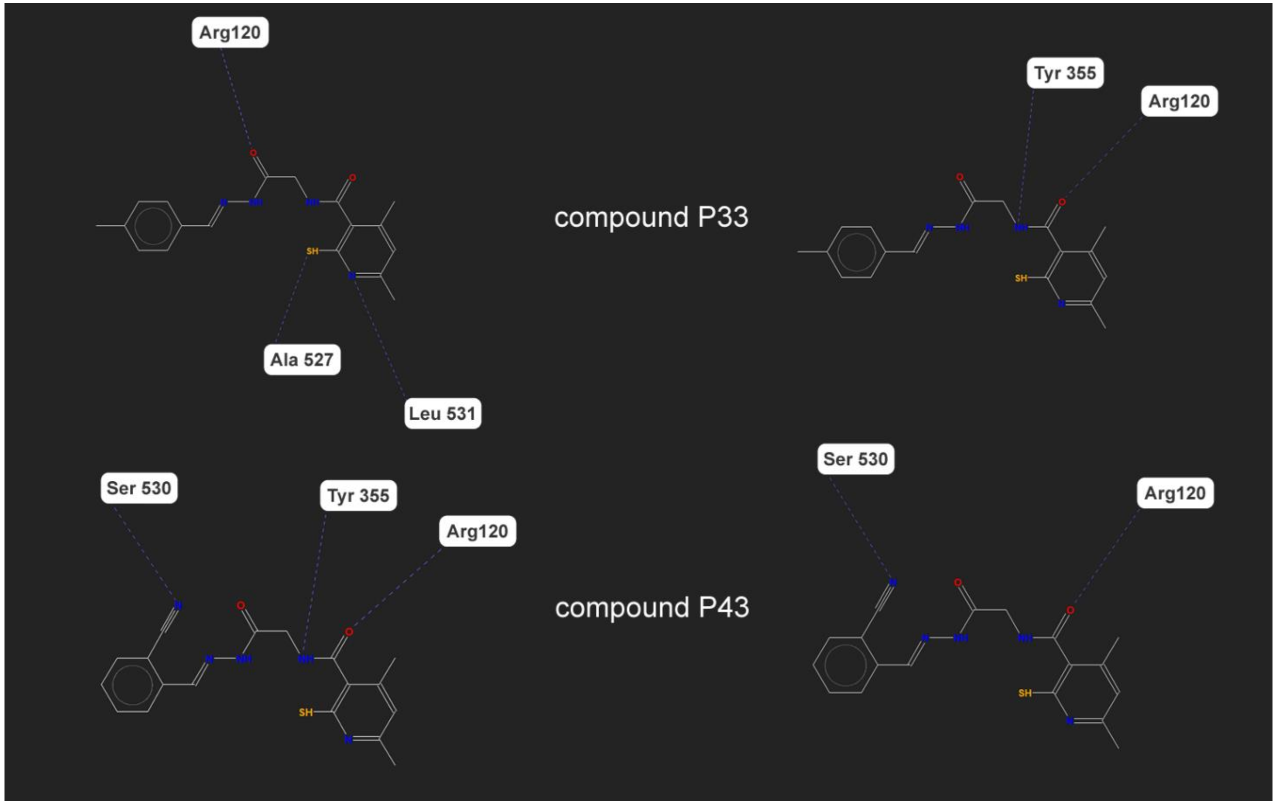

Figure S5. Docking poses of PS33 (blue) and PS43 (magenta) under COX-1 and COX-2 binding domain conditions. Ligand maps representing interactions by hydrogen bonds (blue dot line), details in the main text.
